# Genome –Scale Reconstruction of Metabolic Networks of *Lactobacillus casei* ATCC 334 and 12A

**DOI:** 10.1371/journal.pone.0110785

**Published:** 2014-11-03

**Authors:** Elena Vinay-Lara, Joshua J. Hamilton, Buffy Stahl, Jeff R. Broadbent, Jennifer L. Reed, James L. Steele

**Affiliations:** 1 Department of Food Science, University of Wisconsin-Madison, Madison, Wisconsin, United States of America; 2 Department of Chemical and Biological Engineering, University of Wisconsin-Madison, Madison, Wisconsin, United States of America; 3 DuPont Nutrition and Health, Madison, Wisconsin, United States of America; 4 Utah State University Department of Nutrition, Dietetics, and Food Sciences, Logan, Utah, United States of America; Technical University of Denmark, Denmark

## Abstract

*Lactobacillus casei* strains are widely used in industry and the utility of this organism in these industrial applications is strain dependent. Hence, tools capable of predicting strain specific phenotypes would have utility in the selection of strains for specific industrial processes. Genome-scale metabolic models can be utilized to better understand genotype-phenotype relationships and to compare different organisms. To assist in the selection and development of strains with enhanced industrial utility, genome-scale models for *L. casei* ATCC 334, a well characterized strain, and strain 12A, a corn silage isolate, were constructed. Draft models were generated from RAST genome annotations using the Model SEED database and refined by evaluating ATP generating cycles, mass-and-charge-balances of reactions, and growth phenotypes. After the validation process was finished, we compared the metabolic networks of these two strains to identify metabolic, genetic and ortholog differences that may lead to different phenotypic behaviors. We conclude that the metabolic capabilities of the two networks are highly similar. The *L. casei* ATCC 334 model accounts for 1,040 reactions, 959 metabolites and 548 genes, while the *L. casei* 12A model accounts for 1,076 reactions, 979 metabolites and 640 genes. The developed *L. casei* ATCC 334 and 12A metabolic models will enable better understanding of the physiology of these organisms and be valuable tools in the development and selection of strains with enhanced utility in a variety of industrial applications.

## Introduction

*Lactobacillus casei* are Gram positive, non-sporulating, nutritionally fastidious and strictly fermentative bacteria that produce lactic acid as their principal metabolic end product [1]. *L. casei* strains are used in the manufacture of fermented foods, as probiotics, and has significant potential for the production of biofuels and co-products with a broad range of applications such as food ingredients [2], [3], [4]. The utility of *L. casei* in all of these applications is strain dependent, due to significant variation in gene content that is present in this species. Comparative genome analysis of seventeen *L. casei* strains has revealed this species has an average of 2,800 orthologous genes (±151), of which 1,715 are shared by all the strains, and 4,220 non-conserved orthologous genes with an average of 119 unique genes found in each strain [5]. This large variation in gene content allows for the selection of strains with unique and industrially important characteristics. Therefore, detailed comparisons of strain specific differences at the genomic and phenotypic level are needed.

Genome-scale metabolic models can serve as tools for evaluating the metabolic potential of strains based on genomic data. These models are becoming a common tool for linking genomic data to the biochemical reaction networks that govern cellular processes [6]. They can be utilized to understand genotype-phenotype relationships and to compare behaviors between different organisms [7]. Additionally, genome-scale models can provide a framework for the integration of transcriptomic, proteomic, and metabolomic data, thus offering a unique global view of cellular physiology and microbial responses to environmental and genetic changes. The integration of such datasets can help reduce the multiple possible flux distributions by reducing the solution space. [8]. Genome-scale models are typically analyzed using constraint-based methods, which impose constraints that consider network stoichiometry, thermodynamics, flux capacity and sometimes transcriptional regulation. These constraints limit metabolic flux distributions and cellular phenotypes [9]. Constraint-based models are rapidly becoming essential tools for understanding microbial metabolism and for developing microorganisms for use in a variety of applications. Furthermore, it is now possible to rapidly construct these models using automated reconstruction technologies [8], [10], thereby enabling the adoption of constraint-based models by a larger scientific community.

To date, *in silico* models are available for four species of lactic acid bacteria (LAB): *Lactococcus lactis* [11], [12], *Lactobacillus plantarum* [13], *Streptococcus thermophilus* [14], and *Lactobacillus reuteri* [15]. The first LAB model was used to simulate the shift from homo- to heterolactic fermentation, identify an *in silico* minimal medium and to improve diacetyl production [11]. Additionally, a genome-scale metabolic model for *Lactococcus lactis* MG1363 was developed; this model couples the carbon and nitrogen metabolism of the strain with catabolic pathways that lead to flavor formation in cheese [12]. The genome-scale metabolic reconstruction of *L. plantarum* was successfully used to evaluate nutrient requirements of the WCFS1 strain [13]. The genome-scale metabolic reconstruction of *S. thermophilus* [14] revealed metabolic differences between *S. thermophilus* and other LAB. The most recent LAB genome-scale metabolic models have been developed for three strains of *Lactobacillus reuteri*, the model strain JCM1112, and two human-derived strains, 6475 and 55730; these models were utilized to identify probiotic features of these strains [15]. Genome-scale metabolic models of LAB have allowed for species and strain comparison, provided guidance for metabolic engineering, and stimulated the development of new hypotheses concerning mechanisms by which probiotics survive in hosts. The availability of genome-scale metabolic models for *L. casei* strains would enhance current understanding of how strain specific characteristics influence the utility of these species in various industrial applications.

In this study, genome-scale metabolic models were constructed for two fully sequenced strains of *L. casei*: *L. casei* ATCC 334, a cheese isolate, and 12A a corn silage isolate. A draft genome sequence for 12A was available (GenBank accession number AFYJ00000000) and this genome was closed for the purposes of this study. These models were then utilized to conduct a detailed comparison of these two strains.

## Methods

### Genome Sequencing

Genomic DNA of *L. casei* 12A was prepared using the DNA Isolation Bacterial CTAB protocol as requested by the Department of Energy (DOE) Joint Genome Institute (JGI). This protocol can be found at http://my.jgi.doe.gov/general/protocols.html. Genomic DNA quality was confirmed by agarose gel electrophoresis and was submitted to the Department of Energy Joint Genome Institute for genome sequencing, where Ilumina HiSeq 2000 technology was used. JGI delivered a draft genome containing 397 DNA Scaffolds. This draft genome was assembled in collaboration with DuPont Inc., utilizing a previously available 12A draft genome as a scaffold [5]. The assembly contained 19 contigs with 531 fold coverage. The contigs were ordered using the complete genome of *L. casei* ATCC 334 as a scaffold [16]. The remaining gaps were sequenced at the University of Wisconsin Biotechnology Center.

### Genome Annotation

The complete genome sequence and annotation of *L. cas*ei ATCC 334 (GenBank accession number NC_008526 and NC_008502) [16] and 12A are freely available online at National Center for Biotechnology Information (NCBI) (http://www.ncbi.nlm.nih.gov) and at the RAST Server (Rapid Annotations using Subsystems Technology) (http://rast.nmpdr.org/). The genome sequence for *L. casei* 12A was imported into the RAST server (http://rast.nmpdr.org/) for gene calling and annotation with subsequent manual inspection and curation. This information was used in the metabolic reconstruction and validation processes, as described below.

### Reconstruction of *L. casei* metabolic networks

**Figure 1:**
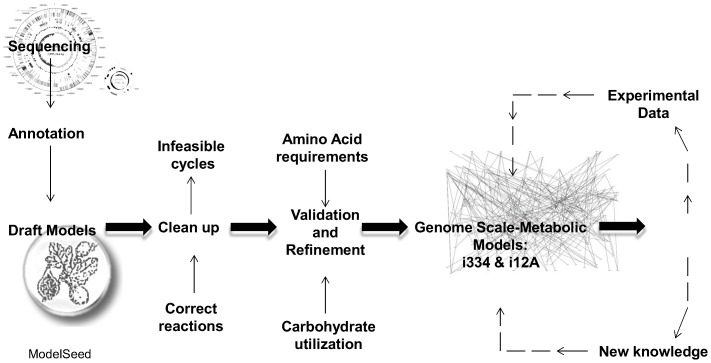
Schematic representation of the approach utilized in the reconstruction of *i*Lca334_548 and *i*Lca12A_640 genome-scale metabolic models. Dashed lines represent iterative processes.

Figure 1 summarizes the overall process of reconstructing the two *L. casei* metabolic networks. Genome-scale metabolic draft models for both *L. casei* strains were generated in 2011 using the RAST server [17] and the Model SEED database [18]. We then identified and removed thermodynamically infeasible cycles (e.g. cycles resulting in free ATP production) [19] and mass- and charge-balanced all reactions. The resulting models were then tested by comparing model predictions to amino acid requirement and carbohydrate utilization data [5]. The models were modified as needed using the SMILEY algorithm [20], which suggests reaction additions from a metabolic reaction database (in this case the Model SEED database) to match growth phenotypes. Reactions from the SEED database were downloaded in 2011, and unbalanced reactions were removed from the database prior to employing SMILEY. A copy of the database used for gapfilling is provided in Table S5 in Excel and in Table S9 in SBML format. Additional reactions not suggested by this algorithm were manually added as needed. Reactions suggested by SMILEY, or manually identified, were only added when sufficient evidence and information was available from experimental data, NCBI, KEGG, JGI, and SEED databases. To compare the metabolic networks we identified orthologous genes between the two genomes, which we defined as bidirectional best-BLAST hits with >90% DNA identity (alignments were run in SEED). A total of 511 pairs of orthologs between the two strains were identified that were included in the models.

### Flux Balance Analysis

Flux balance analysis (FBA) identifies a steady-state metabolic flux distribution that maximizes a stated objective, such as cellular growth rate or product formation [21]. FBA was used to interrogate the properties and capabilities of both *L. casei* metabolic models. FBA simulations were performed by maximizing biomass, with an *in-silico* growth medium containing nutrients in the chemically defined medium (CDM) [22] with additional trace minerals (Table S2). To predict amino acid requirements, each model was allowed uptake of all 20 amino acids. Then, exchange fluxes for each amino acid were individually set to zero and the maximum growth rate was calculated. A maximum growth rate of zero indicated the amino acid was essential. For carbohydrate utilization, only essential amino acids were allowed to be taken up by the models. The set of essential amino acids included arginine, isoleucine, leucine, phenylalanine, tryptophan, tyrosine and valine for both strains, with the addition of glutamate for *L. casei* 12A simulations. We first maximized growth using just the essential amino acids (without a carbohydrate present). We then introduced each carbohydrate to the *in silico* media. An increase in maximum growth rate in the presence of a particular carbohydrate indicated the ability of the model to utilize the carbohydrate as a carbon source.

### Experiments for amino acid requirements

Working cultures were prepared from frozen stocks by two sequential transfers in de Man, Rogosa and Sharpe (MRS) medium and CDM broth prepared as described by Christensen and Steele [22]. Cultures were incubated statically at 37°C for 24 h and 18 h, respectively. To determine experimental amino acid requirements, CDM broth was prepared with the omission of individual amino acids, inoculated with 1% of the working cultures, and incubated at 37°C under static conditions. Optical density at 600 nm was determined at intervals during the 48 h incubation.

### Identification of Functional Network Differences

We used CONGA to identify functional differences between the two metabolic networks [23]. This constraint-based method identifies gene deletions that affect maximum growth rates differently in two models. Simulations were performed under four different growth conditions, varying the carbon source (glucose or galactose) and the amino acids present (all amino acids or essential amino acids). To control for differences in amino acid requirements, glutamate was included as essential amino acid in both models. For each condition, CONGA maximized differences between each model's maximal growth rate when selected gene deletions were imposed (i.e. maximize µ_1_- µ_2_, subject to common gene deletions).

## Results

### Genome sequencing

The closed *L. casei* 12A genome consists of a circular chromosome of 2,907,945 bp. The general features of the 12A genome are presented in Table 1. Compared to *L. casei* ATCC 334, the 12A genome is 12,681 bp larger. The number of pseudogenes in 12A is 152, a relatively high number compared to the 82 in *L. casei* ATCC 334, which could indicate that *L. casei* 12A has undergone more extensive gene decay. Comparative genome analysis between the strains indicates that they share 2,222 orthologous genes; *L. casei* 12A has 507 unique genes compared to *L. casei* ATCC334, whereas *L. casei* ATCC334 has 423 unique genes compared to *L. casei 12A*. The complete genome sequence of 12A was deposited at GenBank with the accession number CP006690.

**Table 1:** Important features of the *L. casei* 12A genome.

Chromosome	
Length, bp	2,907,945
Number of CDs	2,900
G + C content, %	46.44
Genes total number	2,833
Protein Coding Genes	2,746
with function prediction	2,222
without function prediction	524
Number of pseudogenes	152
Number of tRNA genes	57

### Genome-scale metabolic networks

The genome-scale metabolic models of *L. casei* ATCC 334 and 12A each contain an extracellular and cytoplasm compartment, and exchange and transport reactions allow metabolites to be imported into or secreted from these compartments. The model for *L. casei* ATCC 334 (*i*Lca334_548) consists of 548 genes, 905 metabolic reactions and 959 metabolites. The model for *L. casei* 12A (*i*Lca12A_640) consists of 640 genes, 932 metabolic reactions and 979 metabolites. The properties of the models are summarized in Table 2. These models are made available in Table S6 and Table S7 in Excel format and SBML format (Table S10 and Table S11).

**Table 2:** Overview of metabolic models of *L. casei* 12A and ATCC 334

Categories	Strains	
	ATCC 334	12A
**Genes**	548 (37)	640 (129)
**Metabolites**	959 (9)	979 (29)
Intracellular	827(9)	838(20)
Extracellular	132	141(9)
**Reactions**	1040 (23)	1076 (59)
Exchange	133	142(9)
Metabolic	905(22)	932(49)
With +GPRs	818	847
Without GPRs	87	85
*Non-metabolic	2(1)	2(1)

. Numbers in parentheses indicate unique genes, metabolites and reactions present only in one *L. casei* model.

*Non-metabolic reactions include biomass and sink reactions.

+ Gene – Protein- Reactions (GPRs).

### Amino acid requirements

Single amino acid omission experiments in both *L. casei* strains showed the strains have similar amino acids requirements, where the branched-chain amino acids (leucine, isoleucine and valine), the aromatic amino acids (tyrosine, phenylalanine and tryptophan), and arginine are essential for both strains. However, glutamate is essential for *L. casei* 12A growth, while in ATCC 334 the absence of glutamate only caused a growth defect.

Initial draft models from Model SEED were used to predict the amino acid requirements for both strains by FBA. In the case of the ATCC 334 model, 17 out of 20 simulations agreed with the *in vivo* results; the draft model incorrectly predicted tryptophan to be non-essential (false positive) while cysteine and methionine were incorrectly predicted to be essential (false negative). The draft 12A model predictions agreed with the *in vivo* data for 16 of the 20 simulations. The inconsistencies for tryptophan, methionine and cysteine observed with the ATCC 334 model were also observed with the 12A model. In addition, the 12A model predicted that glutamate was non-essential, while the *in vivo* data indicated it was essential. SMILEY [20] was used to fix false negative predictions in the draft models. To fix both model's requirement for cysteine, the algorithm suggested adding reactions carried out by sulfite dehydrogenase (E.C. 1.8.2.1), thiosulfate dehydrogenase (E.C. 1.8.2.2) and cystathionine β-synthase (E.C. 4.2.1.22). Homoserine O-acetyltransferase (E.C. 2.3.1.31) and O-acetylhomoserine sulfhydrylase (E.C. 2.5.1.49) reactions were added to the *L. casei* ATCC 334 model to fix the model's requirement for methionine. While correcting the methionine requirement in the *L. casei* 12A model involved adding methionine synthase (E.C. 2.1.1.13) and O-succinylhomoserine sulfhydrylase (E.C. 4.2.99.9). To correct the discrepancy between the model predictions and the *in vivo* data requirements for tryptophan, some reactions that were initially gap filled by Model SEED (and identified using CytoSEED [24] were removed. The anthranilate synthase (E.C. 4.1.3.21) and tryptophan synthase (E.C. 4.2.1.20) reactions were removed from the 334 and 12A networks, respectively, since there was no evidence to support their inclusion based on genome annotations and experimental data. In the case of the *L. casei* 12A model, it was not possible to fix the discrepancy between the model and *in vivo* data requirement for glutamate since the 12A genome contains a gene (GI:410525452) annotated as glucosamine-fructose-6-phosphate aminotransferase (E.C. 2.6.1.16), which converts glutamine into glutamate. After making these corrections the *L. casei* ATCC 334 model was able to correctly predict the amino acid requirements and the *L. casei* 12A model was able to correctly predict 19 out of the 20 amino acid requirements (Table 3).

**Table 3:** The amino acid requirements of *L. casei* ATCC 334 and 12A as determined by *in vivo* experiments and flux balance analysis of the models prior to and after model refinement.

Amino acids	*L. casei* ATCC 334			*L. casei* 12A		
	*In vivo*	*Draft Model*	*Refined Model*	*In vivo*	*Draft Model*	*Refined Model*
Alanine	NR	NR	NR	NR	NR	NR
Arginine	R	R	R	R	R	R
Asparagine	NR	NR	NR	NR	NR	NR
Aspartate	W	NR	NR	W	NR	NR
**Cysteine**	NR	**R**	NR	NR	**R**	NR
**Glutamate**	W	NR	NR	R	**NR**	**NR**
Glutamine	NR	NR	NR	NR	NR	NR
Glycine	NR	NR	NR	NR	NR	NR
Histidine	NR	NR	NR	NR	NR	NR
Isoleucine	R	R	R	R	R	R
Leucine	R	R	R	R	R	R
Lysine	NR	NR	NR	NR	NR	NR
**Methionine**	NR	**R**	NR	NR	**R**	NR
Phenylalanine	R	R	R	R	R	R
Proline	NR	NR	NR	NR	NR	NR
Serine	NR	NR	NR	NR	NR	NR
Threonine	NR	NR	NR	NR	NR	NR
**Tryptophan**	R	**NR**	R	R	**NR**	R
Tyrosine	R	R	R	R	R	R
Valine	R	R	R	R	R	R
***Accuracy***		***17/20***	***20/20***		***16/20***	***19/20***

Discrepancies between experimental data and simulations are in bold.

R = Amino Acid required for growth (maximal optical density observed ≤0.05).

NR = Amino Acid not required for growth (maximal optical density observed ≥0.15).

W = Weak growth in the absence of the amino acid (maximal optical density observed was 0.09).

### Carbohydrate utilization

**Figure 2:**
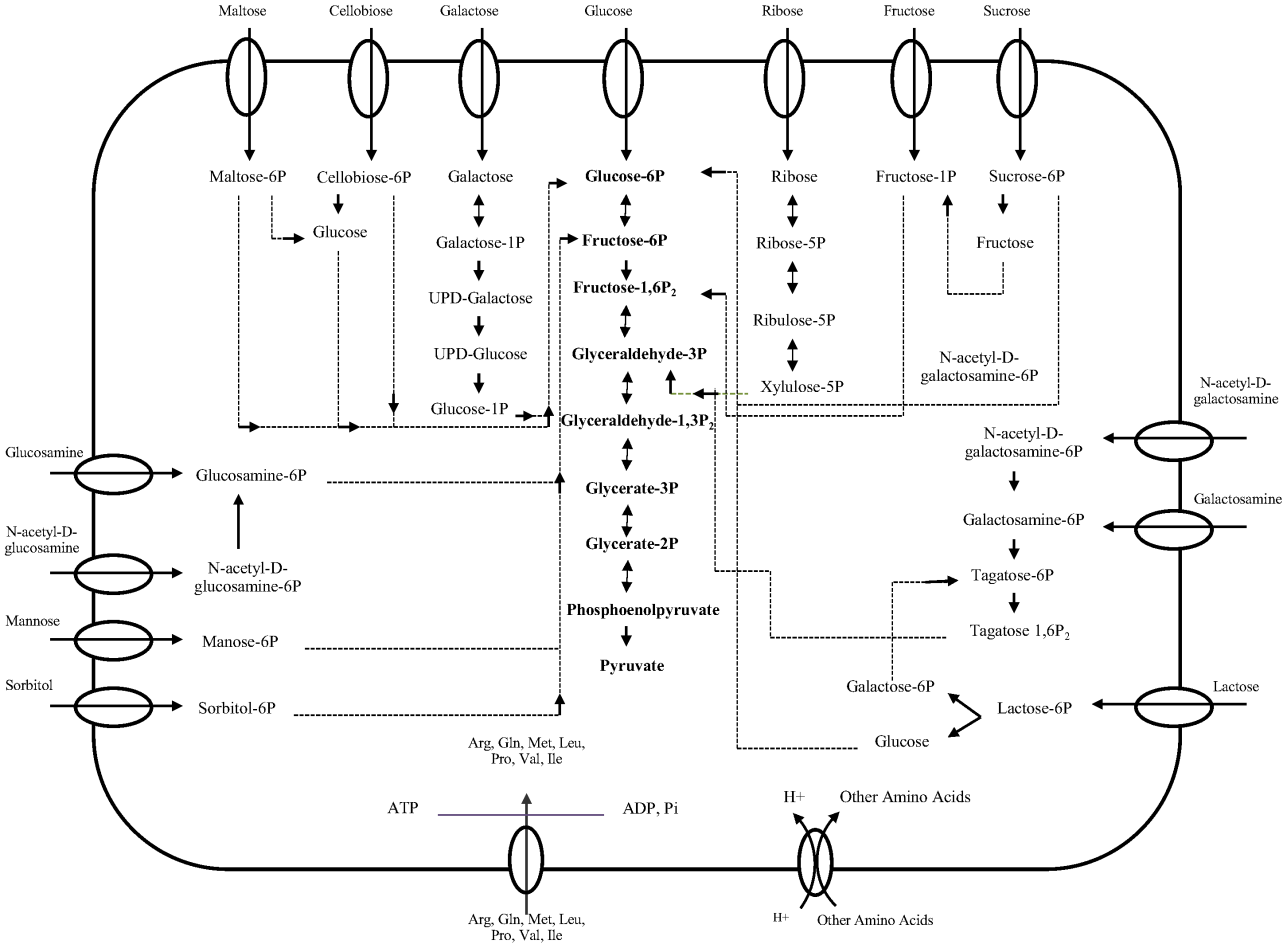
Metabolic map of carbohydrate metabolism of *L. casei* 12A and ATCC 334. Glycolytic metabolites are listed in bold.

Carbohydrate utilization plays a key role in the ecology and industrial utility of *L. casei*; therefore it was important to ensure the models were in agreement with the *in vivo* carbohydrate utilization data. The draft metabolic models of ATCC 334 and 12A were tested against data for utilization of 56 carbohydrates, where the utilization of these carbon sources by ATCC 334 and 12A strains has been reported previously [5]. Both strains were unable to utilize 25 carbohydrates, including β-cyclodextrin, γ-cyclodextrin, amylopectin, amylase, arabinogalactan, carboxymethyl cellulose, D-ribitol, D-arabinose, D-arabitol, dextrin, D-maltitol, D-xylitol, D-xylose, fucose, galacturonic acid, glucuronic acid, heparin, lignin, meso-erythritol, mucin, phytic acid, rhamnose, sialic acid, stachyose, xylan and α-cyclodextrin. For all 25 of these un-usable carbohydrates, the drafts models accurately predicted no growth. The 31 carbohydrates that at least one of the strains could utilize *in vivo* are presented in Table 4. The draft models did not perform as well in predicting use of carbohydrates where growth was observed *in vivo*. The poorer performance of the drafts models was mostly due to missing transporters, likely caused by limitations in carbohydrate transporter annotation, causing these transporters to be missed from draft reconstructions. A total of 7 enzymes and 14 transporters were added to the *L. casei* ATCC 334 model and 7 enzymes and 17 transporters were added to the *L. casei* 12A model to fix false negative predictions (Table S8). For the carbohydrate amygdalin-6P, we were unable to identify a route with genomic evidence to degrade its by-product, mandelonitrile; we introduced a sink reaction to consume this metabolite. A metabolic map of carbohydrate metabolism is presented in Figure 2; this metabolic map indicates how some relevant carbon sources are transported into the cytoplasm and how they are integrated into glycolysis.

**Table 4:** Carbohydrate utilization of *L. casei* ATCC 334 and 12A as determined by *in vivo* experiments and flux balance analysis of the models prior to and after model refinement.

Carbohydrate	*L. casei* ATCC 334			*L. casei* 12A		
	*^+^* *In vivo*	* *Draft Model*	* *Refined Model*	+ *In vivo*	* *Draft Model*	* *Refined Model*
Amygdalin	G	**NG**	G	G	**NG**	G
D-cellobiose	G	G	G	G	G	G
D-fructose	G	**NG**	G	G	**NG**	G
D-galactose	G	**NG**	G	G	**NG**	G
D-glucosamine	G	**NG**	G	G	**NG**	G
D-glucose	G	G	G	G	G	G
D-lactitol	G	**NG**	G	G	**NG**	G
D-maltose	G	G	G	G	G	G
D-mannose	G	G	G	G	G	G
D-melezitose	G	**NG**	G	G	**NG**	G
D-raffinose	NG	NG	NG	G	**NG**	G
D-ribose	G	**NG**	G	G	G	G
D-sorbitol/glucitol	G	**NG**	G	G	G	G
D-turanose	G	**NG**	G	G	**NG**	G
Galactosamine	G	**NG**	G	G	**NG**	G
Gluconic Acid	G	G	G	G	G	G
Inulin	G	**NG**	G	G	**NG**	G
Isomaltose	G	**NG**	G	G	**NG**	G
Lactose	G	**NG**	G	G	**NG**	G
Lactulose	G	**NG**	G	G	**NG**	G
Maltotriose	G	**NG**	G	G	**NG**	G
Myo-inositol	NG	NG	NG	NG	**G**	**G**
N-acetyl-D-galactosamine	G	**NG**	G	G	**NG**	G
N-acetyl-D-glucosamine	G	**NG**	G	G	**NG**	G
Panose	NG	NG	NG	G	**NG**	G
Polydextrose	G	**NG**	G	G	**NG**	G
Pullulan	NG	NG	NG	G	**NG**	G
Sucrose	G	**NG**	G	G	**NG**	G
***Accuracy***		***35/54***	***54/54***		***33/54***	***53/54***

Discrepancies between experimental data and simulations are in bold.

G = Growth in the presence of the carbohydrate.

NG = No growth in the presence of the carbohydrate.

*For simulations, G represents increased biomass production in the presence of the carbohydrate; NG represents no change in biomass production in the presence of the carbohydrate.

+ In vivo data are based on the study cited by Broadbent et al. (2012) [5].

### Identification of Functional Network Differences

CONGA identifies gene deletion sets, sets of one or more genes whose deletion are predicted to be lethal by only one of two models. Gene deletion sets are classified based on the reason for differences in growth predictions: genetic differences, in which gene-protein-reaction (GPR) relationships differ in the models; orthology differences, in which genes encoding enzymes with identical functions were not assigned as orthologs; and metabolic differences, where one organism has additional reactions that grant it unique capabilities [23].

The CONGA results were insensitive to carbon source (indicating there are no differences between glucose or galactose catabolism in the two strains). Therefore, the results are summarized based on amino acid availability. For the first condition (with only essential amino acids), CONGA found five gene deletion sets that were lethal only in *i*Lca334_548 (e.g., no biomass formation was predicted by *i*Lca334_548) and five gene deletion sets that were lethal only in *i*Lca12A_640. For the second condition (all amino acids), two gene deletion sets were lethal only to *i*Lca334_548; and four gene deletion sets were lethal only to *i*Lca12A_640. These deletion sets were a subset of those found in the first condition (where only essential amino acids are present). CONGA results are summarized in Table 5 and the full results are presented in Table S3 and Table S4.

**Table 5:** Number of gene deletion sets found by CONGA under four different conditions between *i*Lca334_548 and *i*Lca12A_640.

Conditions	*Lethal in iLca334_548*	*Lethal in iLca12A_640*
	Genetic = 3	Genetic = 1
Glucose or Galactose + Essential A.A.	Metabolic = 1	Orthology = 4
	Orthology = 1	
Glucose or Galactose + All A.A.	Genetic = 2	Orthology = 4

Four of the ten unique gene deletions sets identified by CONGA were attributed to genetic differences, which arise due to differences in the number of isozymes between the two models for essential reactions. Three of these four gene deletion sets were lethal in *i*Lca334_548. This highlights the presence of more isozymes in *i*Lca12A_640, suggesting it is more robust to gene deletions than *i*Lca334_548.

**Figure 3:**
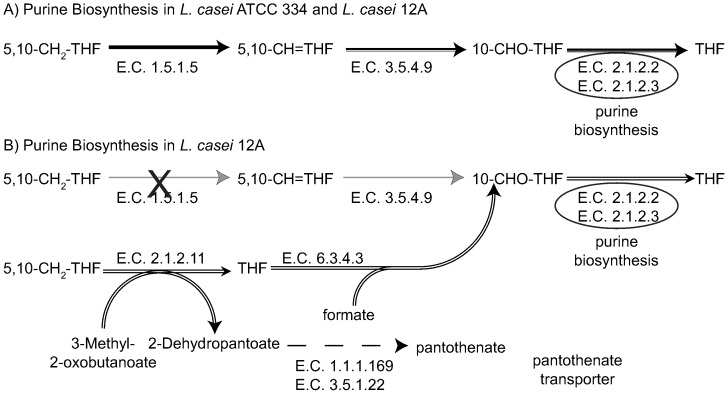
Metabolic differences in the two *L. casei* strains. (A): Pathway for the synthesis of tetrahydrofolate (THF) from 5, 10-methylenetetrahydrofolate (5,10-CH2-THF) and its role in purine biosynthesis. This pathway is common to both strains. (B): Additional pathway for the conversion of 5,10-CH_2_-THF to THF active in the *i*Lca12A_640 model. With the exception of the panthtothenate transporter, the reactions are found in both models. (A and B): Thick arrows indicate flux in both models. Double arrows represent flux in the *i*Lca12A_640 model. The black ‘X’ indicates a gene deletion identified by CONGA lethal in *i*Lca334_548 but not *i*Lca12A_640, and gray arrows indicate inactive reactions arising from the deletion. The dashed arrow represents two separate steps. Reactions and metabolites corresponding to the given E.C. numbers and metabolite identifiers are given in the Supporting Material.

Using CONGA we also identified one metabolic difference, in which deletion of the enzyme 5, 10-methylenetetrahydrofolate (5,10-CH_2_-THF) dehydrogenase (E.C. 1.5.1.5) is lethal only in the *i*Lca334_548 model. This means that the *i*Lca12A_640 model has a unique mechanism (shown in Figure 3) for recovering from this gene deletion. Briefly, 5,10-CH_2_-THF is a precursor to 10-formyltetrahydrofolate (10-CHO-THF) a cofactor involved in purine biosynthesis, an essential activity for cellular growth. The deletion of 5,10-CH_2_-THF dehydrogenase prevents the biosynthesis of THF in *L. casei* ATCC 334. Our model for *L. casei* 12A predicts that this deletion can be rescued by the actions of 5,10-CH_2_-THF:3-methyl-2-oxobutanoate (E.C. 2.1.2.11) and formate: THF ligase (E.C. 6.3.4.3), in which 5,10-CH_2_-THF is converted directly to THF, producing 2-dehydropantoate as a by-product. The reactions pantoate 2-oxidoreductase (E.C. 1.1.1.169) and pantothenate amidohydrolase (E.C. 3.5.1.22) convert 2-dehydropantoate to pantothenate, which can be secreted by a transporter unique to the *i*Lca12A_640 model, or some pantothenate can also be used for CoA biosynthesis, important for biomass formation.

## Discussion

### Genome-scale metabolic networks

In this study, we assembled a draft genome-scale metabolic model for two strains of *L. casei*: ATCC 334 and 12A, using the Model Seed pipeline; followed by model curation to ensure consistent predictions for amino acid requirements and carbohydrate utilization. The *in silico* reconstructions, *i*Lca334_548 and *i*Lca12A_640 appear to have the largest metabolic networks in comparison to other LAB metabolic networks published to date, with 1,040 and 1,076 reactions, respectively.

Our experimental results show one difference in amino acid requirements between the two *L. casei* strains, glutamate is essential for *L. casei* 12A but not in *L. casei* ATCC334. However, differences between the strains are more apparent based on their carbohydrate utilization.

### Amino acid requirements

Compared to other LAB with reported metabolic models, our strains have fewer amino acid requirements than some *Lactobacillus* species (e.g. *L. reuteri* JM112 and *L. plantarum* WCFS1). On the other hand, our strains have considerably more requirements than *Lc. lactis* and *S. thermophilus* (Table S1). The rich environments from which our *Lactobacillus* strains were isolated reduce the need for synthesizing amino acids.

The branched-chain amino acid (isoleucine, leucine and valine) biosynthesis pathways in *L. casei* ATCC 334 and 12A appear to be absent based on genome annotation, which was confirmed by the growth experiments indicating their essentiality.

Aromatic amino acids (tryptophan, phenylalanine and tyrosine) had incomplete biosynthesis pathways in the two *L. casei* strains. In the biosynthesis pathway of tryptophan, the enzyme anthranilate synthase (E.C. 4.1.3.27) was not found in the genome annotation of *L. casei* ATCC 334 strain; presumably this reaction was added to the draft metabolic models as a mean to complete the pathway. Experimental data indicating tryptophan must be supplied support the idea that this pathway is incomplete. In *L. casei* 12A the reaction catalyzed by tryptophan synthase (E.C. 4.2.1.20) was found to confer the model, the ability to synthesize tryptophan, therefore it was removed base on experimental data. Since tryptophan was required for growth by both strains, a reaction was deleted from both metabolic models. The requirement for phenylalanine and tyrosine for growth was expected, as an enzyme needed for their biosynthesis, prephenate dehydratase, was not found in the genome annotation of either strain.

*L. casei* strains are reported to require arginine for growth [25]; therefore a requirement for the amino acid was expected in both cases. The enzyme arginine dihydrolase (E.C. 3.5.3.6), involved in arginine biosynthesis, was not found in the genome annotation of either strain.

The omission of glutamate in the medium caused a severe defect in growth for *L. casei* ATCC 334 and no growth for *L. casei* 12A. This has been well documented in other LAB, due to an incomplete citric acid cycle and limited supply of α-ketoglutarate [26], but the metabolic models in both cases predicted growth in the absence of this amino acid. This can be explained by the presence of glutamine. In both metabolic models glutamine can be converted into glutamate using the reaction catalyzed by glucosamine-fructose-6-phosphate aminotransferase (E.C. 2.6.1.16). When this enzyme is deleted from *i*Lca12A_640, glutamate is predicted to be an essential amino acid, however, this deletion would also make glutamine a predicted essential amino acid, but glutamine is not required experimentally, therefore the reaction was not removed. Experimental studies suggest that the interconversion of glutamine to glutamate results in low yields of glutamate [27], therefore glutamate is likely needed even in the presence of glutamine.

### Carbohydrate utilization

Comparison of the two *L. casei* models highlights the presence of additional enzymes that gives the *L. casei* 12A model the ability to utilize D-rafinnose, panose and pullulan. For instance, the 12A model possesses an ABC transporter predicted to function for raffinose uptake. This model also includes a pullulanase and a neopullulanase, important in pullulan and panose degradation into maltotriose and maltose and glucose, respectively. As discussed by Broadbent *et al.* (2012), the ability of strains to utilize different carbohydrates predicts the adaptation of the strains to diverse ecological niches; D-rafinnose, panose and pullulan are components commonly found in plant material, the environment from which *L. casei* 12A was isolated. On the other hand, as observed in growth experiments and confirmed by the metabolic models, *L. casei* ATCC 334, can utilize fewer of the carbohydrates tested (see Table 4). This could be related to evolution of cheese isolates by gene decay [5], suggesting that dairy environments are richer in less complex carbon sources than those found in plant environments.

One of the most abundant stereoisomers of inositol is *myo*-inositol (MI), a component of phytic acid, which can serve as a phosphate storage molecule in plant seeds [28]. Most LAB species cannot utilize this carbon source, although Yebra *et al*. (2007) reported the identification of a gene cluster which enables *L. casei* BL23 to utilize myo-inositol under aerobic conditions. The catabolic pathway of MI is absent in *L. casei* ATCC 334, and experimental studies and prediction by the metabolic models confirmed no growth on MI. Surprisingly, the genome-scale metabolic model for *L. casei* 12A predicted growth using MI as a carbon source, implying the catabolic pathway for MI is complete in this strain. Further analysis in the genome annotation of *L. casei* 12A reveals the presence of all the enzymes (E.C. 1.1.1.18, 4.2.1.44, 3.7.1, 5.3.1, 2.7.1.92, 4.1.2.29 and 5.3.1.1) needed for converting MI to glyceraldehyde-3P. The environment from which the 12A strain was isolated could have played a role in the retention of the pathway. The reason why *L. casei* 12A was unable to utilize MI is speculated to be due to regulatory effects that genome-scale metabolic models do not capture.

### Identification of Functional Network Differences

A unique transporter (pantothenate sodium symporter) in *i*Lca12A_640 for pantothenate was found to confer the model with the ability to overcome the deletion of 5,10-CH_2_-THF dehydrogenase. This transporter prevents the accumulation of pantothenate inside the cell, therefore allowing the reactions, catalyzed by the enzymes E.C. 2.1.2.11 and 6.3.4.3 to be active, yielding to the synthesis of 10-CHO-THF.

The pantothenate transporter in *i*Lca12A_640 (added by Model SEED) lacks a gene association, however further analysis of *L. casei* 12A genome annotation reveals the presence of a pantothenate energy-coupling factor (ECF) transporter (GI 410523916). An ortholog of this gene was found in *L. casei* ATCC 334, which is annotated as a predicted membrane protein which may be the reason it was missed during the automated reconstruction of the model. This provides an example of how CONGA can be used to aide in further model development.

CONGA simulations performed under the glucose and galactose conditions, both six carbon carbohydrates, identified the same differences between the models. Simulations with ribose where also conducted (data not shown), but no new gene deletions sets were found. Even though we were able to identify genetic, orthology and metabolic differences between the models, it appears that pathways in catabolism of these three sugars and central metabolism are shared by the strains.

The genome-scale metabolic models of two *L. casei* strains provide a comprehensive overview of the metabolism of the strains. These models are valuable tools for the development of strains with enhanced utility in a variety of industrial applications (e.g. food ingredients), as well as in the design of new metabolic engineering approaches in the production of commodity chemicals such as biofuels.

## Supporting Information

Table S1 **Comparison of essential amino acids (listed as E) determined from experiments among lactic acid bacteria, for which metabolic models have been built.**
(DOCX)

Table S2 **Compounds allowed to be taken up during flux balance analysis in both** ***L. casei*** **models.** Flux represents uptake rate, units are mmol/gDW/h.
(DOCX)

Table S3 **Type of gene deletion sets, with glucose or galactose with essential amino acids condition, found in** ***i*** **Lca12A_640 and** ***i*** **Lca334_548.**
(DOCX)

Table S4 **Type of gene deletion sets, with glucose or galactose and all amino acids, found in** ***i*** **Lca12A_640 and** ***i*** **Lca334_548.**
(DOCX)

Table S5 **Complete list of reactions and compounds present in GapfillMatrix.**
(XLSX)

Table S6 **Complete list of reactions and compounds present in iLca12A_640.**
(XLSX)

Table S7 **Complete list of reactions and compounds present in iLca334_548.**
(XLSX)

Table S8 **Enzymes and transporters added to** ***L. casei*** **ATCC 334 and 12A models to fix false negative predictions.** Addition of an enzyme/transporter is indicated with filled circles; open circles indicate no addition of enzyme/transporter.
(DOCX)

Table S9 **GapfillMatrix in SBML format.**
(XML)

Table S10 ***i*** **Lca12A_640 in SBML format.**
(XML)

Table S11 ***i*** **Lca334_548 in SBML format.**
(XML)
