# Supplementary material for: Genome –Scale Reconstruction of Metabolic Networks of Lactobacillus casei ATCC 334 and 12A
Source: PLoS One. 2014 Nov 3;9(11):e110785. doi: 10.1371/journal.pone.0110785 (PMC4231531; doi:10.1371/journal.pone.0110785)
Supplement: Table S1 — Comparison of essential amino acids (listed as E) determined from experiments among lactic acid bacteria, for which metabolic models have been built. (DOCX) [file pone.0110785.s001.docx]

Table S1. Comparison of essential amino acids (listed as E) determined from experiments among lactic acid bacteria, for which metabolic models have been built.

|  | **Arg** | **Leu** | **Ile** | **Val** | **Tyr** | **Trp** | **Phe** | **Glu** | **Met** | **Thr** | **His** | **Asn** | **Gln** | **Cys** |
| --- | --- | --- | --- | --- | --- | --- | --- | --- | --- | --- | --- | --- | --- | --- |
| *L. casei* ATCC 334 | **E** | **E** | **E** | **E** | **E** | **E** | **E** |  |  |  |  |  |  |  |
| *L. casei* 12A | **E** | **E** | **E** | **E** | **E** | **E** | **E** | **E** |  |  |  |  |  |  |
| *L. reuteri JCM1112* | **E** | **E** |  | **E** | **E** | **E** | **E** | **E** | **E** | **E** | **E** | **E** | **E** |  |
| *L. plantarum* WCFS1 | **E** | **E** | **E** | **E** | **E** | **E** | **E** | **E** | **E** | **E** |  |  |  | **E** |
| *L. lactis* MG1363 |  | **E** |  |  |  |  |  | **E** | **E** |  | **E** |  |  |  |
| *S. thermophilus* LMG18311 |  |  |  |  |  |  |  |  |  |  | **E** |  |  | **E** |
