# Supplementary material for: Genome –Scale Reconstruction of Metabolic Networks of Lactobacillus casei ATCC 334 and 12A
Source: PLoS One. 2014 Nov 3;9(11):e110785. doi: 10.1371/journal.pone.0110785 (PMC4231531; doi:10.1371/journal.pone.0110785)
Supplement: Table S2 — Compounds allowed to be taken up during flux balance analysis in both L. casei models. Flux represents uptake rate, units are mmol/gDW/h. (DOCX) [file pone.0110785.s002.docx]

**Table S2. Compounds allowed to be taken up during flux balance analysis in both *L. casei* models. Flux represents uptake rate, units are mmol/gDW/h.**

| **Compound** | **Name** | **Max. Uptake Flux** |
| --- | --- | --- |
| *Components of CDM* |  |  |
| cpd00030 | Manganese (Mn) | 1000 |
| cpd00048 | Sulfate (SO4) | 1000 |
| cpd00063 | Calcium (Ca) | 1000 |
| cpd00099 | Chlorine (Cl) | 1000 |
| cpd00205 | Potassium (K+) | 1000 |
| cpd00220 | Riboflavin | 1000 |
| cpd00254 | Magnesium (Mg) | 1000 |
| cpd00305 | Thiamin | 1000 |
| cpd00393 | Folate | 1000 |
| cpd00971 | Sodium ( Na) | 1000 |
| cpd00001 | Water (H2O) | 1000 |
| cpd00009 | Phosphate | 1000 |
| cpd00011 | Carbon dioxide (CO2) | 1000 |
| cpd00067 | Hydrogen (H) | 1000 |
| Carbon source | variable | 5 |
| Amino acids | variable | 0.9 |
| *Trace Minerals and others* |  |  |
| cpd00034 | Zn | 1000 |
| cpd00058 | Cu | 1000 |
| cpd00149 | Co | 1000 |
| cpd10516 | Fe | 1000 |
| cpd00264 | Spermidine | 1000 |
