# Supplementary material for: Genome –Scale Reconstruction of Metabolic Networks of Lactobacillus casei ATCC 334 and 12A
Source: PLoS One. 2014 Nov 3;9(11):e110785. doi: 10.1371/journal.pone.0110785 (PMC4231531; doi:10.1371/journal.pone.0110785)
Supplement: Table S4 — Type of gene deletion sets, with glucose or galactose and all amino acids, found in i Lca12A_640 and i Lca334_548. (DOCX) [file pone.0110785.s004.docx]

**Table S4. Type of gene deletion sets, with glucose or galactose and all amino acids, found in *i*Lca12A_640 and *i*Lca334_548**

| ***i*Lca12A_640 Number** | ***i*Lca12A_640 Genes** | ***i*Lca334_548Number** | ***i*Lca334_548 Genes** | ***i*Lca12A_640 Growth** | ***i*Lca12A_640 Production** | ***i*Lca334_548 Growth** | ***i*Lca334_548 Production** | **Objective** | ***i*Lca12A_640 Reactions** | ***i*Lca334_548 Reactions** | **Type of gene set deletion** | **Explanation** |
| --- | --- | --- | --- | --- | --- | --- | --- | --- | --- | --- | --- | --- |
| 1 | peg2232 | 1 | peg1926 | 1 | 1 | 0 | 0 | 1 |  | rxn00917 | Genetic | *i*Lca12A_640 has one isoenzyme for rxn00917 |
| 1 | peg1097 | 1 | peg106 | 1 | 1 | 0 | 0 | 1 |  | rxn01644 | Genetic | *i*Lca12A_640 has 3 isoenzymes for rxn01644 |
| 1 | peg2706 | 0 |  | 0 | 0 | 1 | 1 | 1 | rxn05177 |  | Orthology |  |
| 1 | peg1651 | 0 |  | 0 | 0 | 1 | 1 | 1 | rxn05645 |  | Orthology |  |
| 1 | peg470 | 0 |  | 0 | 0 | 1 | 1 | 1 | rxn00346 |  | Orthology |  |
| 1 | peg2452 | 0 |  | 0 | 0 | 1 | 1 | 1 | rxn00555 |  | Orthology |  |
