# Supplementary material for: Genome –Scale Reconstruction of Metabolic Networks of Lactobacillus casei ATCC 334 and 12A
Source: PLoS One. 2014 Nov 3;9(11):e110785. doi: 10.1371/journal.pone.0110785 (PMC4231531; doi:10.1371/journal.pone.0110785)
Supplement: Table S8 — Enzymes and transporters added to L. casei ATCC 334 and 12A models to fix false negative predictions. Addition of an enzyme/transporter is indicated with filled circles; open circles indicate no addition of enzyme/transporter. (DOCX) [file pone.0110785.s008.docx]

| **Table S8. Enzymes and transporters added to *L. casei* ATCC 334 and 12A models to fix false negative predictions. Addition of an enzyme/transporter is indicated with filled circles; open circles indicate no addition of enzyme/transporter.** | | | | |
| --- | --- | --- | --- | --- |
| **Enzyme Name/Transporter** | **E.C. Number** | **Reactions** | *i*Lca334_548 | *i*Lca12A_640 |
| β-Glucosidase/β-Fructosidase | 3.2.1.21\|3.2.1.14 | rxnadd9,rxnadd10,rxnadd12,rxnadd13,rxnadd16,rxnadd24, rxnadd26, rxn00816, rxn14329 | ● | ● |
| D-sorbitol-6-phosphate:NAD 2-oxidoreductase | 1.1.1.140 | rxn03886 | ● | ● |
| ATP:D-glucosamine phosphotransferase | 2.7.1.11 | rxn01439 | ● | ● |
| ATP:D-fructose 1-phosphotransferase | 2.7.1.3 | rxn00627 | ● | ● |
| ATP:N-acetyl-D-glucosamine 6-phosphotransferase | 2.7.1.59 | rxn00892 | ● | ● |
| N-Acetyl-D-galactosamine 6-phosphate amidohydrolase | 3.5.1.25 | rxn03504 | ● | ● |
| α-D-Glucose 1-phosphate 1,6-phosphomutase | 5.4.2.2\|5.4.2.5 | rxn00704 | ● | ● |
| Acetate transport in/out via proton symport | NA | rxn5488 | ● | ● |
| Amygdalin | NA | rxnadd8 | ● | ● |
| Citrate uptake | NA | rxn 5211 | ● | ● |
| D-Galactosamine uptake | NA | rxnadd1 | ● | ● |
| D-Galactose ABC transport | NA | rxn05162 | ● | ● |
| Indole transport via proton symport, reversible | NA | rxn10176 | ● | ○ |
| Inulin uptake | NA | rxnadd15 | ● | ● |
| Isomaltose | NA | rxnadd6 | ● | ● |
| Lactitol uptake | NA | rxnadd23 | ● | ● |
| Lactulose uptake | NA | rxnadd25 | ● | ● |
| Melezitose ABC Transport System | NA | rxnadd11 | ● | ● |
| Panose uptake | NA | rxnadd2 | ● | ● |
| Polydextrose uptake | NA | rxnadd17 | ● | ○ |
| Turanose uptake | NA | rxnadd14 | ● | ● |
| Lactose uptake | NA | rxnadd4 | ○ | ● |
| Lactose transport via PEP:Pyr PTS | NA | rxn10865 | ○ | ● |
| Panose uptake | NA | rxnadd19 | ○ | ● |
| Pullulan uptake | NA | rxnadd21 | ○ | ● |
| Raffinose uptake | NA | rxnadd5 | ○ | ● |
